# Female sex is a risk factor for painful diabetic peripheral neuropathy: the EURODIAB prospective diabetes complications study

**DOI:** 10.1007/s00125-023-06025-z

**Published:** 2023-10-23

**Authors:** Jackie Elliott, Gordon Sloan, Lynda Stevens, Dinesh Selvarajah, Giorgio Cruccu, Rajiv A. Gandhi, Peter Kempler, John H. Fuller, Nishi Chaturvedi, Solomon Tesfaye, for the EURODIAB Prospective Complications Study Group

**Affiliations:** 1 Diabetes Research Unit, Royal Hallamshire Hospital, Sheffield, UK; 2 Department of Oncology and Metabolism, University of Sheffield, Sheffield, UK; 3 Department of Epidemiology and Public Health, University College, London, UK; 4 Department of Neurological Sciences, La Sapienza University, Rome, Italy; 5 First Department of Medicine, Semmelweis University, Budapest, Hungary; 6 Epidemiology and Public Health, Imperial College of Science, Technology & Medicine, London, UK; 7 MRC Unit for Lifelong Health & Ageing at UCL, Institute of Cardiovascular Sciences, University College London, London, UK

**Keywords:** Diabetic peripheral neuropathy, Epidemiology, Neuropathic pain, Painful diabetic neuropathy, Painful neuropathy, Type 1 diabetes mellitus

## Abstract

**Aims/hypothesis:**

While the risk factors for diabetic peripheral neuropathy (DPN) are now well recognised, the risk factors for painful DPN remain unknown. We performed analysis of the EURODIAB Prospective Complications Study data to elucidate the incidence and risk factors of painful DPN.

**Methods:**

The EURODIAB Prospective Complications Study recruited 3250 participants with type 1 diabetes who were followed up for 7.3±0.6 (mean ± SD) years. To evaluate DPN, a standardised protocol was used, including clinical assessment, quantitative sensory testing and autonomic function tests. Painful DPN (defined as painful neuropathic symptoms in the legs in participants with confirmed DPN) was assessed at baseline and follow-up.

**Results:**

At baseline, 234 (25.2%) out of 927 participants with DPN had painful DPN. At follow-up, incident DPN developed in 276 (23.5%) of 1172 participants. Of these, 41 (14.9%) had incident painful DPN. Most of the participants who developed incident painful DPN were female (73% vs 48% painless DPN *p*=0.003) and this remained significant after adjustment for duration of diabetes and HbA_1c_ (OR 2.69 [95% CI 1.41, 6.23], *p*=0.004). The proportion of participants with macro- or microalbuminuria was lower in those with painful DPN compared with painless DPN (15% vs 34%, *p*=0.02), and this association remained after adjusting for HbA_1c_, diabetes duration and sex (*p*=0.03).

**Conclusions/interpretation:**

In this first prospective study to investigate the risk factors for painful DPN, we definitively demonstrate that female sex is a risk factor for painful DPN. Additionally, there is less evidence of diabetic nephropathy in incident painful, compared with painless, DPN. Thus, painful DPN is not driven by cardiometabolic factors traditionally associated with microvascular disease. Sex differences may therefore play an important role in the pathophysiology of neuropathic pain in diabetes. Future studies need to look at psychosocial, genetic and other factors in the development of painful DPN.

**Graphical Abstract:**

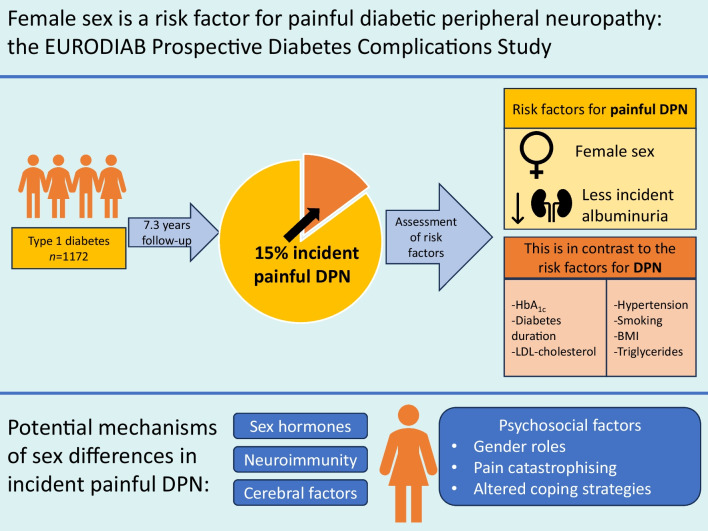


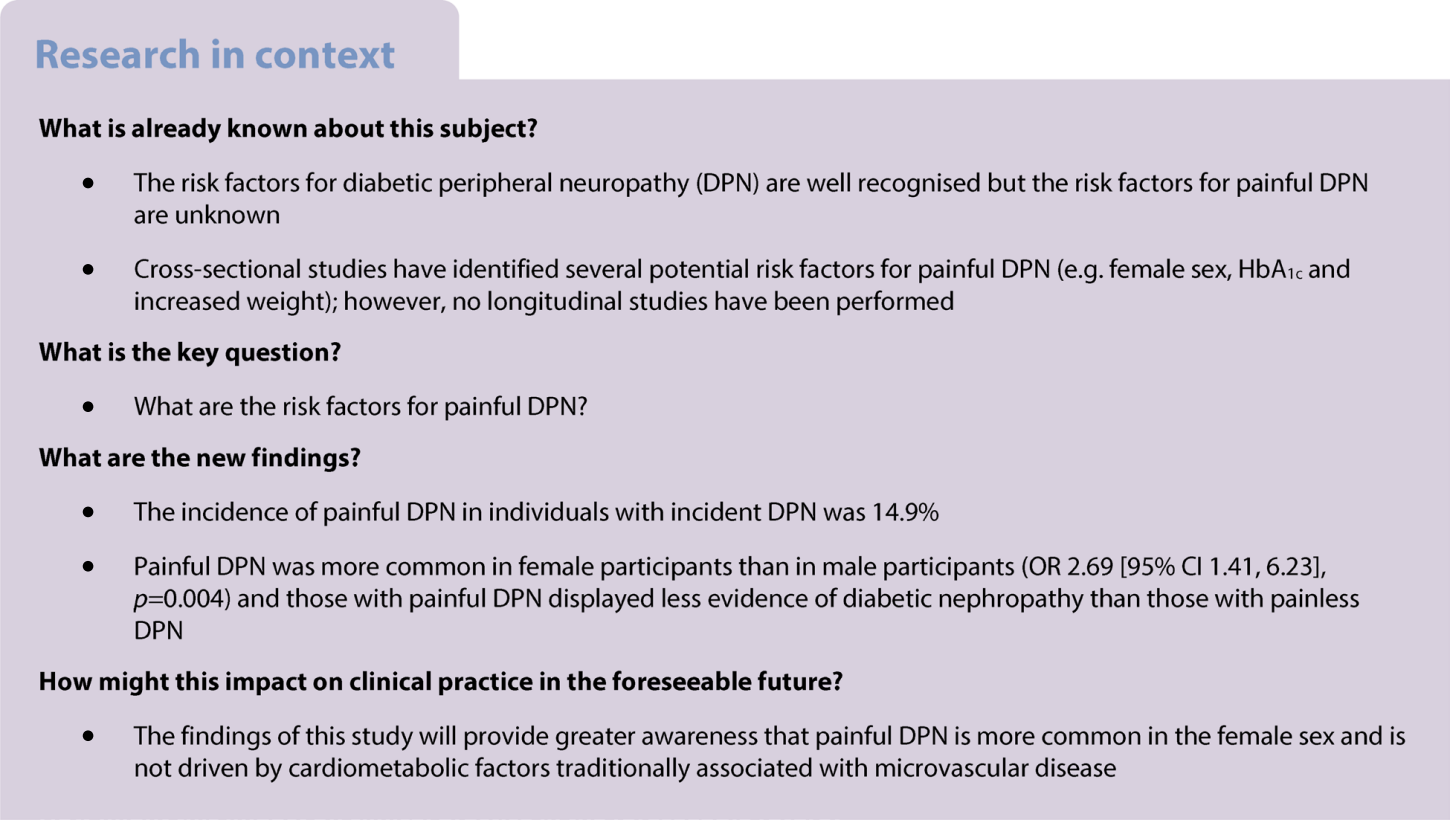


## Introduction

Painful diabetic peripheral neuropathy (painful DPN) affects up to 25% of individuals with diabetes and is a leading factor that prompts those with DPN to seek medical advice [1, 2]. Painful DPN presents with a range of neuropathic symptoms, including burning, deep aching, pins and needles and electric shock like pains, resulting in moderate to severe unremitting lower-limb pain in over 70% of cases [3, 4]. These unpleasant symptoms have a profound effect on sufferers’ lives, leading to insomnia [4], poor quality of life [4, 5], unemployment [6] and depression [6, 7]. Unfortunately, current treatments for painful DPN are only partially effective at best, providing 50% pain relief in less than 50% of affected individuals [8]. Moreover, individuals with painful DPN have significant healthcare resource utilisation costs [9]. A recent study from the USA found that annual direct medical costs for painful DPN were over double those for painless DPN, and over four times those for diabetes alone [9]. There is therefore a good rationale for a better understanding of the risk factors for this disease as it might give insight into new treatment and prevention strategies.

The risk factors for DPN have been extensively studied in high-quality prospective studies [10, 11]. In the EURODIAB study that followed 1172 participants with type 1 diabetes who did not have DPN at baseline, the incidence of DPN was found to be 23.5% over 7.3±0.6 years [11]. The risk factors for incident DPN included duration of diabetes, elevated HbA_1c_, obesity, smoking, elevated triglycerides, elevated urinary AER and hypertension. Conversely, there is no consensus on the risk factors of painful DPN, although a number of factors have been proposed, including age, obesity, duration of diabetes and female sex [3, 5, 12–22]. However, all the studies investigating the risk factors for painful DPN are cross-sectional and therefore not as robust as those examining the risk factors for DPN. The EURODIAB Prospective Diabetes Complications Cohort thus represents a unique opportunity to investigate the risk factors for painful DPN in a prospective study.

## Methods

### Participants and baseline investigations

The EURODIAB Prospective Complications Study recruited 3250 patients (1668 men and 1582 women; sex was determined by participant self-report; no-one reported a different sex from those assigned at birth) with type 1 diabetes from 31 clinics across Europe; the study was therefore representative of the local European population of type 1 diabetes patients. Participant selection and the EURODIAB Prospective Complications Study methodology have been described in detail previously [11, 23]. In brief, participants were selected at random from lists stratified by 5 year age groups and sex; therefore, there was adequate age and sex representation. Physicians were trained in all standardised procedures and baseline examinations were conducted from 1989 to 1991. A subsequent follow-up visit occurred between 1997 and 1999, with a mean ± SD follow-up period of 7.3±0.6 years. Clinical history (including medication history, past medical history and alcohol intake) and examination, participant morphometric and biochemical data were collected at baseline. Baseline biochemical blood tests (HbA_1c_, von Willebrand factor, fibrinogen, lipids, vitamin B_12_, folate, thyroid function, liver function tests) and urine sampling (24 h urinary excretion rate from a single 24 h urine collection) were performed as previously described [11]. The study received approval from ethics committees at each centre and written informed consent was gained from all participants.

### Assessment of nephropathy and retinopathy

Microalbuminuria was defined as a urinary AER of 20–200 μg/min and macroalbuminuria was defined as a rate greater than 200 μg/min. The presence and severity of diabetic retinopathy was determined from centrally graded retinal photographs taken with a wide-angle camera (two fields per eye) [24]. Diabetic retinopathy was classified as either background/non-proliferative or proliferative. Cardiac autonomic neuropathy (CAN) was assessed by the change in systolic BP and the electrocardiographic RR ratio on standing after participants had rested for 5 min in a supine position [11]. The presence of CAN was defined as a loss of heart rate variability, RR ratio <1.04 of the longest RR interval between 28th and 32nd beats after standing and the shortest interval between the 13th and 17th beats [11].

### Assessment and definition of DPN

Physicians underwent central training in London and all clinical examinations and investigations (including autonomic function and vibration perception threshold [VPT] tests) were standardised. Participants with history, examination or biochemical features suggesting other forms of diabetic neuropathy (e.g. diabetic amyotrophy) or polyneuropathy (e.g. vitamin B_12_ deficiency or chemotherapy induced) due to causes other than diabetes were excluded. Assessment of VPT was measured by centrally calibrated biothesiometers (Bio-medical Instrument Company, Newbury, OH, USA). Three readings on the right big toe and right medial malleolus were obtained and averaged. Results were classified according to age-related criteria [25].

DPN was defined as the presence of two or more of the following criteria [11]: the presence of one or more neuropathic symptoms (defined as any of the following in the preceding 6 months: ‘asleep’ numbness or ‘dead feeling’ in the feet; a ‘prickling’ sensation in the feet; deep aching pains in the legs and/or feet; burning pains in the legs and/or feet; unusual difficulty in climbing stairs; difficulty with bladder control, and nocturnal diarrhoea); the absence of two or more reflexes of the ankle or knee tendons (with reinforcement if necessary); an abnormal VPT; and the presence of CAN. In those participants with incident DPN, the presence of painful DPN was assessed. Painful DPN was deemed present if a participant had evidence of distal symmetrical polyneuropathy (according to the protocol) with painful neuropathic symptoms (deep aching or burning pains) in the distribution of the peripheral neuropathy.

### Statistical analysis

Data were analysed using the statistical package SAS 9.2 (SAS Institute, NC, USA). Measured baseline risk factors were compared between people with incident painless and painful DPN at follow-up. For data that were parametric, group means were compared using the Student’s *t* test. For non-parametric data, medians were compared using the Mann–Whitney *U* test. The χ^2^ test was used to assess differences between group percentages. Multiple logistic regression was used to adjust for confounding factors (HbA_1c_ and duration of diabetes) and to calculate standardised ORs. For continuous risk factors, this is the change in odds associated with an increase of 1 SD in that risk factor and for dichotomous variables the standardised OR has as a reference group those participants without the respective risk factor. A *p* value lower than 0.05 was considered statistically significant.

## Results

### Incidence of painful DPN

**Fig. 1:**
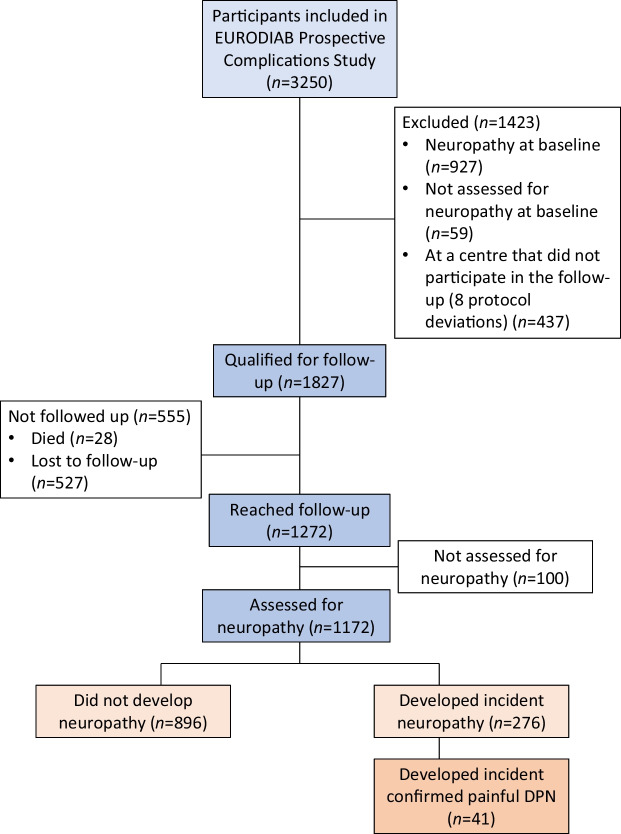
Participants examined for progression to painful neuropathy in the EURODIAB study [11]

As previously described, 3250 individuals were examined at baseline; 3193 were assessed for neuropathy, of which 927 were diagnosed as having DPN [11]. The prevalence of painful DPN at baseline in those with DPN was 25.2% (234/927). At follow-up, 1172 participants without DPN at baseline were reassessed (Fig. 1). DPN had developed in 276 participants, giving an incidence of 23.5% [11]. Incident painful DPN was reported in 41 out of these 276 patients (14.9%).

### Risk factors of painful DPN

The baseline characteristics of the participants who went on to develop painful and painless DPN are presented in Table 1. Comparing the participants with painless and painful DPN, there were no significant differences in clinical and demographic variables, including age, BMI, HbA_1c_, lipid profile and BP. However, the proportion of female participants was significantly higher in the incident painful DPN (73%) compared with painless DPN group (48%, *p*=0.003). Height was lower in the painful DPN group, which could be accounted for by the higher proportion of female participants. In those that developed painful DPN, the degree of nephropathy at baseline was less, with lower rates of micro- or macroalbuminuria (*p*=0.02). However, there was no difference in the severity of peripheral neuropathy between the two groups, as measured by VPT, CAN or number of abnormal criteria used to define DPN.

**Table 1:** Baseline characteristics of 276 participants according to the incidence of painful or painless diabetic neuropathy

Variable	Painless DPN (*n*=235)	Painful DPN (*n*=41)	*p* value
Age, years	33.4±9.9	35.0±10.1	0.3
Duration of diabetes, years	14.9±8.9	15.0±9.0	0.9
Sex, % female	48	73	0.003**
History of smoking, %	52	63	0.2
Height, cm	169±9.1	166±9.0	0.04*
Weight, kg	69.1±11.3	67.4±10.2	0.4
BMI, kg/m^2^	24.1±3.0	24.5±3.2	0.5
WHR	0.84±0.11	0.81±0.11	0.07
HbA_1c_, mmol/mol	68±15.4	69±13.0	0.7
HbA_1c_, %	8.4±1.9	8.5±1.6	
Insulin, U/kg body weight	0.67±0.20	0.69±0.22	0.5
Total cholesterol, mmol/l	5.43±1.16	5.56±0.90	0.4
LDL-cholesterol, mmol/l	3.44±1.00	3.48±0.93	0.8
HDL-cholesterol, mmol/l	1.47±0.43	1.50±0.36	0.7
Triglyceride, mmol/l	0.93 (0.54, 3.10)	1.14 (0.57, 2.51)	0.1
Fibrinogen, g/l	3.16±0.85	3.21±1.14	0.7
von Willebrand factor, U/ml	1.20 (0.63, 2.28)	1.23 (0.53, 1.91)	0.9
Systolic BP, mmHg^a^	119 (97, 154)	115 (78,152)	0.2
Diastolic BP, mmHg^a^	75 (57, 89)	76 (48,95)	0.9
Hypertension, %	26	20	0.4
History of CVD, %	12	17	0.4
AER, μg/min	11.9 (3.8, 464.0)	9.9 (3.1, 155.7)	0.1
Macroalbuminuria, %	8.3	2.7	0.2
Micro- or macroalbuminuria, %	34	15	0.02*
Any retinopathy, %	52	48	0.5
Proliferative retinopathy, %	8	7	0.8
CAN, %	26	29	0.6

Data are presented as percentages or means ± SD or, for non-normal distribution, as medians (5th percentile, 95th percentile)

^a^The BP data exclude participants who were undergoing antihypertensive therapy

^*^p<0.05, **p<0.01

To further explore the risk factors for incident painful DPN, ORs were calculated. As higher HbA_1c_ levels and duration of diabetes are known to increase the incidence of DPN [11] and thus act as confounders, they were adjusted for in this analysis (Table 2). A lower proportion of participants with incident painful DPN had micro- or macroalbuminuria. The participants with painful DPN also had a lower calculated AER. In addition, those with painful DPN had lower WHR, were shorter and, most striking of all, were 2.7 times more likely to be female. As lower height and smaller WHR are associated with female sex, the ORs were calculated adjusting for sex, as well as HbA_1c_ and duration of diabetes (Table 2). Neither height nor WHR were statistically significant in participants with painful DPN in this analysis. However, the proportion of individuals displaying micro- or macroalbuminuria remained significantly lower and the AER remained significantly lower. To examine whether this association is a risk factor for incident painful neuropathy in both men and women, a sex-stratified analysis was carried out (Table 3). The AER was still significantly lower in female participants with incident painful DPN, but not in male participants.

**Table 2:** Risk factors for painful neuropathy after adjustment for HbA_1c_ and duration of diabetes and for HbA_1c_, duration of diabetes and sex

Variable	OR (95% CI)	*p* value
Adjustment for HbA_1c_ and duration of diabetes		
Female sex	2.69 (1.41, 6.23)	0.004
Height, cm	0.70 (0.49, 0.99)	0.04
WHR	0.69 (0.46, 1.03)	0.07
AER, μg/min^a^	0.59 (0.30, 0.95)	0.03
Micro- or macroalbuminuria, %	0.34 (0.13, 0.88)	0.03
Adjustment for HbA_1c_, duration of diabetes and sex		
Height, cm	0.98 (0.61, 1.56)	0.9
WHR	0.91 (0.62, 1.34)	0.6
AER, μg/min^a^	0.60 (0.36, 1.00)	0.05
Micro- or macroalbuminuria, %	0.35 (0.13, 0.91)	0.03

Standardised ORs are expressed per SD increase in each continuous risk factor

ORs for dichotomous variables have as a reference group those participants without the respective risk factor

^a^Log transformation was used

**Table 3:** Risk factors for painful neuropathy after adjustment for HbA_1c_ and diabetes duration by sex

Variable	OR (95% CI)	*p* value
Men		
Height, cm	0.99 (0.51, 1.90)	0.9
WHR	0.70 (0.31, 1.56)	0.4
AER, μg/min^a^	0.87 (0.39, 1.94)	0.7
Micro- or macroalbuminuria, %	0.22 (0.03, 1.87)	0.2
Women		
Height, cm	0.96 (0.63, 1.46)	0.9
WHR	0.98 (0.65, 1.49)	0.9
AER, μg/min^a^	0.53 (0.30, 0.93)	0.03
Micro- or macroalbuminuria, %	0.39 (0.13, 1.17)	0.09

Standardised ORs are expressed per SD increase in each continuous risk factor. ORs for dichotomous variables have as a reference group those participants without the respective risk factor

^a^Log transformation was used

## Discussion

Chronic painful DPN can be extremely distressing and is a leading cause of morbidity and healthcare utilisation [1, 3–7, 9]. Furthermore, its pathophysiology remains undetermined and current treatments provide sub-optimal pain relief [8, 26]. Therefore, there is a clear rationale for identifying risk factors for painful DPN. Despite this, few well-designed studies have looked at the incidence and risk factors for painful DPN and in particular there have been no prospective studies. Thus, the EURODIAB Prospective Diabetes Complication Study, one of the largest multi-centre prospective diabetes studies, provides a unique opportunity. The prevalence of painful DPN in a cohort of 3250 individuals (mean age 30.7 years) at baseline was 7.2%. After excluding participants with DPN at baseline, 276 (23.5%) developed DPN after 7.3 years, and of these 14.9% had incident painful DPN. In this European study, there was a striking preponderance for the development of painful DPN in female participants, providing the strongest evidence so far for female sex being a major risk factor for painful DPN. Furthermore, there was less evidence of nephropathy at baseline, measured by albuminuria, in participants with incident painful compared with painless DPN. This suggests that the development of painful DPN may not be driven by simple cardiometabolic factors but may be influenced by psychological, social, cultural, genetic and other factors [2, 7, 22]. Although there have been cross-sectional studies this is the first prospective study to show that female sex is a risk factor for painful DPN.

Key risk factors for DPN, including poor glycaemic control and markers of large vessel disease, such as hypertension, smoking, increased triglycerides, obesity and raised cholesterol have already been reported in the same cohort of participants [11]. Similar findings have since been confirmed more recently [10] in type 2 diabetes. In contrast, the risk factors for painful DPN are less well known [2]. However, a number of cross-sectional studies have found increasing age [12, 14, 15, 19], duration of diabetes [12, 15, 19, 27], obesity [12–14, 16, 27] and severity of neuropathy [5, 16–18] to be risk factors for painful DPN. Female sex has also been highlighted as a potential risk factor for painful DPN in recent studies [3, 15, 17, 19–21, 27, 28]. Nevertheless, it has been highlighted that prospective epidemiological studies are needed to confirm that female sex is a risk factor for painful DPN [29]. Our study meets this need and proves the findings from cross-sectional studies, including a large cohort study in England (*N*=15,692) that found women had a 50% increased adjusted risk for painful DPN compared with men [3]. More recently, Truini et al consecutively enrolled 816 diabetes patients and found that 13% had painful DPN, with female sex as the only identifiable risk factor (*p*=0.03 vs painless DPN) [20]. Another recent study also demonstrated that female participants with diabetes reported a higher frequency and intensity of pain despite milder nerve injury [29]; however, other studies [3, 19] that assessed pain severity did not find a significant relationship with female sex. It is noteworthy that all these previous studies were cross-sectional and that our large and well-conducted European study definitively demonstrates a causal link between female sex and painful DPN.

There is now a considerable body of literature that suggests there is a difference between men and women in the prevalence of chronic pain [30]. Population-based research has consistently demonstrated a greater prevalence of chronic pain conditions, including neuropathic pain, among women relative to men [31–33]. However, the mechanisms underlying sex differences in chronic pain are incompletely understood. Sex hormones are known to contribute to sexual differentiation of the nervous system and are hypothesised to be involved in pain modulation [34]. Fluctuations in oestrogen levels may contribute to increased pain sensitivity in women whereas testosterone in men may promote pain relief. Sex hormones also appear to interact with the neuroimmune system to alter sensory neuron activity. Recent animal model data indicate different innate and adaptive immune system responses to neuropathic pain models between males and females [35]. The involvement of microglia and T cells in mediating pain hypersensitivity appears to be sexually dimorphic, whereas macrophages, primary sensory neurons and spinal dorsal horn neurons are involved in a sex-independent manner [35]. A recent review concluded that the use of transcriptomic analysis for studying neuropathic pain could be an unbiased, effective strategy to identify molecular mechanisms and better therapeutic targets in men and women [35]. Moreover, significant sex-specific cerebral differences have been demonstrated not only in neuropathic pain but also in chronic pain. In women, key regions of the brain responsible for detection and processing of nociception (e.g. primary somatosensory cortex, insular cortex, anterior cingulate cortex and thalamus) have been found to have altered structure and function, and response to experimental pain [36]. Furthermore, it is possible that sex differences in pain are not entirely of a biological basis as several psychosocial and cultural factors have also been proposed, including sociocultural differences in gender roles, higher levels of catastrophising and altered coping strategies [30, 33]. Clearly the pathophysiological basis of sex differences in pain requires urgent attention, as this could have a considerable impact upon the prevention and treatment of neuropathic pain in women. Moreover, although several studies have demonstrated a higher prevalence of painful DPN in women, it is not known whether there are specific sex differences in the pathophysiology of painful DPN [37]. Clearly, well-designed mechanistic research in well-characterised (phenotyped) individuals is required to investigate this further.

In this study, incident painful DPN was not related to clinical or metabolic factors but it was associated with a lower baseline prevalence of diabetic nephropathy [11]. We know that DPN (painless) is driven by glycaemic control and traditional risk factors for CVD [11]. We also know that the development of nephropathy is similarly driven by cardiometabolic factors [38], hence the increased baseline prevalence of albuminuria in those that develop painless DPN. The lack of relationship between cardiometabolic factors and the incidence of painful DPN suggests that the development of neuropathic pain appears to be more complex and may not be entirely explained by cardiometabolic factors. After all, pain is well recognised to be influenced by cultural, environmental and psychosocial factors in addition to potential factors including peripheral structural/molecular biomarkers [37], central nervous system pain processing [37], genetics [22] and sex [20].

Our study provides the only prospective incidence data for painful DPN. In this cohort of young individuals with type 1 diabetes (mean age 30.7 years), we found the incidence of painful DPN to be 14.9% in those with confirmed DPN after 7 years of follow-up. Additionally, the baseline prevalence of painful DPN in our study was 25.2%. This is relatively similar to the prevalence reported by studies conducted in individuals with type 1 diabetes and type 2 diabetes [3], and type 2 diabetes [1, 5]. However, the reported prevalence rates for painful DPN varies greatly among studies (5.8–54.8%) [1, 3, 5, 12, 13]. The predominant reason for this is the differences in case definition and diagnostic techniques used among studies, although population differences may also contribute. A strength of our study is the robust detection of painful DPN, using the presence of common neuropathic pain symptoms in the presence of confirmed DPN using neurophysiological tests [39]. We therefore believe that our incidence and prevalence data are valid.

The great strengths of this study are that it is a large, prospective study with several years of follow-up and that participants underwent comprehensive neuropathy and diabetes evaluation. Moreover, the study was performed in participants with type 1 diabetes, who were younger with potentially fewer confounding factors than people with type 2 diabetes. However, it may have some limitations. This study is an analysis of the EURODIAB cohort, with the participant follow-up completed in 1999. Since then, there have been improvements in neurological and pain phenotyping (e.g. calf skin biopsy [intra-epidermal nerve fibre density] and modern detailed quantitative sensory testing) that were not available when the study was designed. Additionally, participants unrepresentative of local European ethnic groups were not recruited into the study, which may have an impact on generalisability of the study findings. Moreover, neuropathic pain severity was not assessed in this epidemiological study. However, we believe the results are valid, due to the study being well-designed and representing the only prospective study to identify risk factors of painful DPN.

In conclusion, this largest-ever prospective study of painful DPN in type 1 diabetes, including over 1100 individuals followed for 7 years, has shown the strongest evidence that women are more at risk of developing painful DPN. Incident painful DPN was not related to metabolic variables (glycaemic control, hyperlipidaemia, etc.) or advanced microvascular disease but was associated with lower incidence of albuminuria. The findings of this study will provide greater awareness that female patients are at risk of developing painful DPN in clinical practice.

## Data Availability

Datasets generated during the current study are available from the corresponding author on reasonable request.

## Abbreviations

CAN: Cardiac autonomic neuropathy
DPN: Diabetic peripheral neuropathy
VPT: Vibration perception threshold

## Authors’ relationships and activities

ST reports having received research support from Procter and Gamble and Viatris, and receipt of honoraria of consulting fees from Nevro, Bayer, Worwag Pharma, Angelini and Mitsubishi Tanabe Pharma. ST also reports participation in company sponsored speaker’s bureau for Viatris, Novo Nordisk, Pfizer, Merck, Eva Pharma, Hikma, Grunenthal, Astellas Pharma, Abbott, AstraZeneca, Berlin-Chemie, P&G, Worwag Pharma, Nevro and Haisco Pharmaceutical Group. The other authors declare that there are no relationships or activities that might bias, or be perceived to bias, their work.

## EURODIAB Prospective Complications Study investigators

In addition to the authors, the following investigators participated in the EURODIAB Prospective Complications Study: B. Karamanos, A. Kofinis and K. Petrou (Hippokration Hospital, Athens, Greece); F. Giorgino, G. Picca, A. Angarano, G. De Pergola, L. Laviola and R. Giorgino (Endocrinologia e Malattie Metaboliche, DETO, Università Degli Studi di Bari, Bari, Italy); M. Songini, A. Casu, M. Pedron, S. Pintus and M. Fossarello (Ospedale San Michele, Cagliari, Italy); J. B. Ferriss, G. Grealy and D. O. Keefe (Cork Regional Hospital, Cork, Ireland); M. Toeller and C. Arden (Heinrich-Heine University, Dusseldorf, Germany); R. Rottiers, C. Tuyttens and H. Priem (University Hospital of Gent, Belgium); P. Ebeling, M. Kylliäinen and V. A. Koivisto (University Hospital of Helsinki, Finland); B. Idzior-Walus, J. Sieradzki and K. Cyganek (Jagiellonian University, Krakow, Poland); H. H. P. J. Lemkes and M. Krans (Leiden University Medical Centre, the Netherlands); J. Nunes-Correa, M. C. Rogado, L. Gardete-Correia, M. C. Cardoso, A. Silva, J. Boavida and M. Machado Sa Marques (Portuguese Diabetic Association, Lisbon, Portugal); G. Michel, R. Wirion and S. Cardillo (Centre Hospitalier, Luxembourg); G. Pozza, R. Mangili and V. Asnaghi (Ospedale San Raffaele, Milan, Italy); E. Standl, B. Schaffler, H. Brand and A. Harms (City Hospital Schwabing, Munich, Germany); B. Soussan, O. Verier-Mine, P. Fallas and M. C. Fallas (Centre Hospitalier de Valenciennes, France); J. H. Fuller, J. Holloway, L. Asbury and D. J. Betteridge (University College, London, UK); G. Cathelineau, A. Bouallouche and B. Villatte Cathelineau (Hospital Saint-Louis, Paris, France); F. Santeusanio, G. Rosi, V. D'Alessandro, C. Cagini, P. Bottini and P. Reboldi (Instituto di Patologia Medica, Policlinico, Perugia, Italy); R. Navalesi, G. Penno, S. Bandinelli, R. Miccoli and M. Nannipieri (Dipartimento di Endocrinologia e Metabolismo, Pisa, Italy); G. Ghirlanda, C. Saponara, P. Cotroneo, A. Manto and A. Minnella (Università Cattolica del Sacro Cuore, Rome, Italy); J. D. Ward, S. Tesfaye, S. Eaton and C. Mody (Royal Hallamshire Hospital, Sheffield, UK); M. Borra, P. Cavallo Perin, S. Giunti, G. Grassi, G. F. Pagano, M. Porta, R. Sivieri, F. Vitelli and D. Ferrari (Dipartimento di Medicina Interna, Università di Torino and ASO CTO-CRF Maria Adelaide, Turin, Italy); N. Papazoglou and C. Manes (General Hospital of Thessaloniki, Greece); M. Muggeo and M. Iagulli (V Cattedra di Malattie del Metabolismo, Verona, Italy); K. Irsigler and H. Abrahamian (Hospital Vienna Lainz, Austria); S. Walford, J. Sinclair, S. Hughes, V. McLelland and J. Ward (New Cross Hospital, Wolverhampton, UK); G. Roglic, Z. Metelko, Z. R. Pepeonik and Z. Babic (Vuk Vrhovac Institute for Diabetes, Zagreb, Croatia); and C. Ionescu-Tirgoviste, A. Coszma and C. Guja (Clinic of Diabetes, Nutrition & Metabolic Diseases, Bucharest, Romania).

**Co-ordinating centre** J. H. Fuller, N. Chaturvedi, J. Holloway, D. Webb, L. Asbury, M. Shipley and S. J. Livingstone, University College London, London, UK.

**Central laboratories** G.-C. Viberti, R. Swaminathan, P. Lumb, A. Collins and S. Sankaralingham, Guy’s and St Thomas Hospital, London, UK.
